# Comparative Evaluation of Different Chitosan Species and Derivatives as Candidate Biomaterials for Oxygen-Loaded Nanodroplet Formulations to Treat Chronic Wounds

**DOI:** 10.3390/md19020112

**Published:** 2021-02-15

**Authors:** Monica Argenziano, Bruno Bressan, Anna Luganini, Nicole Finesso, Tullio Genova, Adriano Troia, Giuliana Giribaldi, Giuliana Banche, Narcisa Mandras, Anna Maria Cuffini, Roberta Cavalli, Mauro Prato

**Affiliations:** 1 Department of Drug Science and Technology, University of Turin, 10125 Turin, Italy; monica.argenziano@unito.it; 2 Department of Neuroscience, University of Turin, 10126 Turin, Italy; brexanb@gmail.com; 3 Department of Oncology, University of Turin, 10126 Turin, Italy; nicole.finesso@unito.it (N.F.); giuliana.giribaldi@unito.it (G.G.); 4 Department of Life Sciences and Systems Biology, University of Turin, 10123 Turin, Italy; anna.luganini@unito.it; 5 Department of Surgical Sciences, University of Turin, 10126 Turin, Italy; tullio.genova@unito.it; 6 Istituto Nazionale di Ricerca Metrologica (INRIM), 10135 Torino, Italy; a.troia@inrim.it; 7 Department of Public Health and Pediatric Sciences, University of Turin, 10126 Turin, Italy; giuliana.banche@unito.it (G.B.); narcisa.mandras@unito.it (N.M.); annamaria.cuffini@unito.it (A.M.C.)

**Keywords:** nanodroplets, oxygen, chitosan, chitosan-derivatives, chronic wound, human keratinocytes

## Abstract

Persistent hypoxia is a main clinical feature of chronic wounds. Intriguingly, oxygen-loaded nanodroplets (OLNDs), filled with oxygen-solving 2H,3H-decafluoropentane and shelled with polysaccharides, have been proposed as a promising tool to counteract hypoxia by releasing a clinically relevant oxygen amount in a time-sustained manner. Here, four different types of chitosan (low or medium weight (LW or MW), glycol-(G-), and methylglycol-(MG-) chitosan) were compared as candidate biopolymers for shell manufacturing. The aim of the work was to design OLND formulations with optimized physico-chemical characteristics, efficacy in oxygen release, and biocompatibility. All OLND formulations displayed spherical morphology, cationic surfaces, ≤500 nm diameters (with LW chitosan-shelled OLNDs being the smallest), high stability, good oxygen encapsulation efficiency, and prolonged oxygen release kinetics. Upon cellular internalization, LW, MW, and G-chitosan-shelled nanodroplets did not significantly affect the viability, health, or metabolic activity of human keratinocytes (HaCaT cell line). On the contrary, MG-chitosan-shelled nanodroplets showed very poor biocompatibility. Combining the physico-chemical and the biological results obtained, LW chitosan emerges as the best candidate biopolymer for future OLND application as a skin device to treat chronic wounds.

## 1. Introduction

Chronic wounds, including bedsores, burns, and diabetes-associated vasculopathies, are commonly defined as nonhealing breaks in the epithelial continuity of the skin lasting for more than 42 days [1]. Notably, chronic wounds have become in the last decades an alarming emergency, affecting a large fraction of the population worldwide and the elderly especially, with 1–2% of the population of developed countries being estimated to experience at least one chronic wound during a lifetime [2]. Therefore, the search and the development of new cost-effective treatments that might allow a significant percentage of chronic wounds to heal quickly and definitively appears extremely urgent, as it would substantially affect the global healthcare system [3].

From a physio-pathological perspective, these nonhealing lacerations are generally characterized by persistent hypoxia, exacerbated inflammation, and unbalanced levels between proteinases and their inhibitors, which are essential to accomplish the re-modeling phase of healing [4,5,6]. In addition, microbial infection can represent a recurrent severe complication [7]. In particular, the management of extreme hypoxia in chronic wounds represents a major limit to overcome, and oxygen supply and tension in the wound bed are both crucial issues that need to be addressed in order to promote physiological wound healing. A minimum tissue oxygen tension of 20 mmHg is required for a wound to heal; however, oxygen tension runs at around 5 mmHg in nonhealing wounds [8,9]. Notably, when the arterial blood cannot supply cells with relevant amounts of oxygen, the risks for inflammation, infection, and scarring in wounds are enhanced, since responses to hypoxia are virtually involved in all wound healing and re-modeling processes, including collagen deposition, epithelialization, fibroplasia, angiogenesis, and resistance to infections [8,9]. Another important issue revolves around medical therapy, as the effectiveness of treatments often decreases while tissue necrosis can develop in hypoxic conditions [9]. Oxygen tissue levels can be adequately bolstered by hyperbaric oxygen therapy (HBOT) [10], while an alternative therapy is represented by topical oxygen therapy (TOT) [11]. Unfortunately, either HBOT or TOT display various backwards. On the one hand, the HBOT approach is relatively expensive, uneasy, and even dangerous due to fire accident risks [12]. Additionally, it may cause severe side effects, including myopia, brain toxicity associated with seizures, and pneumothorax [13]. On the other hand, TOT―which is cheaper and associates with lower toxicity―often fails to effectively trespass the stratum corneum of the skin and to deliver oxygen adequately to fibroblasts, keratinocytes, and inflammatory cells for restoring their functions [12]. For these reasons, intensive research has been focused in the recent years on the development of new oxygen carriers, including hemoglobin-based carriers and perfluorocarbon-containing formulations [14]. Among perfluorocarbon emulsions of the first generation, Fluosol^®^ is the only medical device approved by the Food and Drugs Administration and unfortunately no perfluorocarbon-based oxygen emulsion of the second generation is currently approved for clinical uses [15].

In this context, nanomedicine has become a major area of interest for oxygen delivery due to its many unique characteristics [16,17,18,19]. Interestingly, the physico-chemical properties of nanocarriers, including their high surface-to-volume ratio, small size, stability, and controlled drug release enhance their chances to trespass barriers such as the skin and to reach the biological target [20]. In addition, several nanocarriers can be endowed with an inherent antimicrobial activity, thus synergizing with the antimicrobial efficacy of a coupled antibiotic [21]. All these findings appear extremely intriguing, since they may be exploited to produce innovative and nonconventional nanotherapies for wound management [22,23]. 

In particular, over the last decade our collaborating interdisciplinary research network has been working to develop new platforms of oxygen carriers to be employed either for diagnostic or for therapeutic purposes, leading to the production of so-called oxygen-loaded microbubbles (OLMBs) [24], nanobubbles (OLNBs) [25,26], and nanodroplets (OLNDs) [27,28,29,30,31]. Structurally, these carriers display a typical core/shell architecture. For the manufacturing of the outer shell, several alternative polysaccharides (chitosan or dextran usually) can be employed, whereas specific oxygen-solving fluorocarbons (perfluoropentane (PFP) for OLMBs or OLNBs, and 2H,3H-decafluoropentane (DFP) for OLNDs) have been selected for the inner core. 

Among all three platforms, to date OLNDs are likely to represent the more effective oxygen-releasing carrier to be potentially employed for the treatment of hypoxia-related pathologies. Indeed, the DFP peculiar structure allows oxygen molecules to solubilize not only through van der Waals forces, as for PFP, but also by establishing hydrogen intermolecular bonds [32]. As a consequence, DFP incorporates biatomic oxygen more effectively than PFP while its release by diffusion appears slower and sustained over time [27,28]. Nevertheless, nanodroplets still conserve all the peculiar characteristics of nanobubbles, including nanometric size, surface charge, stability, biocompatibility, and responsiveness to ultrasounds (US) [27,28]. The latter property appears crucial, since it allows the nanocarriers to trespass biological barriers through sonophoresis [33]. Interestingly, US irradiation was reported to induce DFP vaporization [34], thus promoting the transformation of nanodroplets into nanobubbles and allowing them to undergo cavitation and sonophoresis. This is consistent with either in vitro or in vivo data showing US-dependent effective trespassing of skin layers by nanodroplets [27,28,35,36] along with higher and more time-sustained oxygen release from oxygen-loaded nanodroplets (OLNDs) with respect to oxygen-free nanodroplets (OFNDs) [27,28]. Due to all these properties, chitosan OLNDs have been proposed as potential therapeutic tools to treat hypoxia-associated pathologies, including chronic wounds, preeclampsia, and cancer. In this context, a series of studies were conducted to investigate the effects of hypoxia and OLNDs on the regulation of cellular secretion of inflammation- and matrix degradation-related soluble factors. Encouragingly, chitosan OLNDs proved to be able to abrogate the dysregulating effects induced by hypoxia, promoting a normoxia-like phenotype in all the hypoxic cells or tissues under investigation [37,38]. Moreover, chitosan-shelled OLND have been shown to be effective against methicillin-resistant *Staphylococcus aureus* (MRSA) and *Candida albicans* infections―two recurring complicating agents in chronic wounds―due to chitosan natural antimicrobial properties [35].

To further optimize chitosan OLND effectiveness for the potential treatment of chronic wounds, in the present work we aimed at investigating different types of chitosan as a candidate biopolymer to be used as OLND shell’s component. A series of chitosan OLNDs such as low weight (LW), medium weight (MW), glycol-(G-), or methylglycol-(MG-) chitosan-shelled OLNDs were prepared and in vitro characterized comparing their physico-chemical properties, efficacy in oxygen releasing, and biocompatibility with human skin cells.

## 2. Results

### 2.1. Characterisation of Chitosan-Shelled ND Formulations

**Figure 1:**
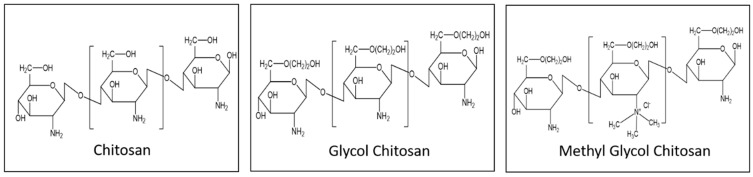
Chemical structure of chitosan (low weight (LW-), medium weight (MW-c)), glycol chitosan (GC-c), methylglycol chitosan (MG-c).

ND formulations were prepared by employing different types of chitosan, such as LW, MW, G-, and MG-chitosan. Figure 1 showed the chemical structure of the different types of chitosan used.

After manufacturing, chitosan-coated oxygen-loaded nanodroplet (cOLND) formulations were characterized physico-chemically by TEM, and dynamic light scattering analyses. The physico-chemical characteristics of cOLNDs are reported in Table 1.

**Figure 2:**
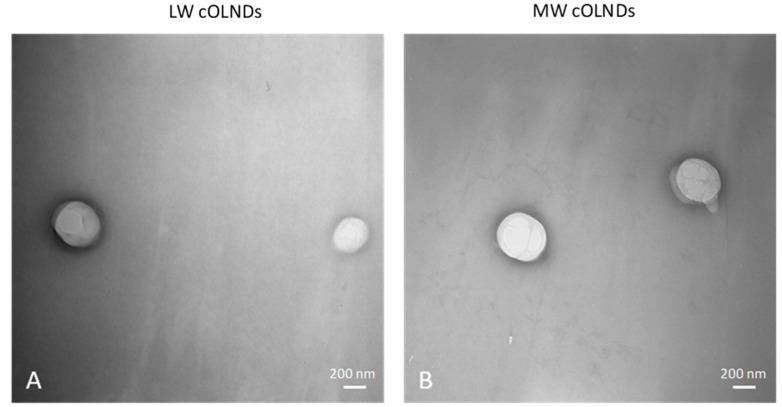
TEM images displaying chitosan-shelled oxygen-loaded nanodroplets (OLND) spherical morphology and shell-core structure. LW and MW cOLNDs were comparatively analyzed by TEM. Results are shown as representative images from ten different preparations. (**A**) LW cOLNDs. (**B**) MW cOLNDs. Magnification: 28,500×.

Figure 2 shows representative images of cOLND morphology.

**Figure 3:**
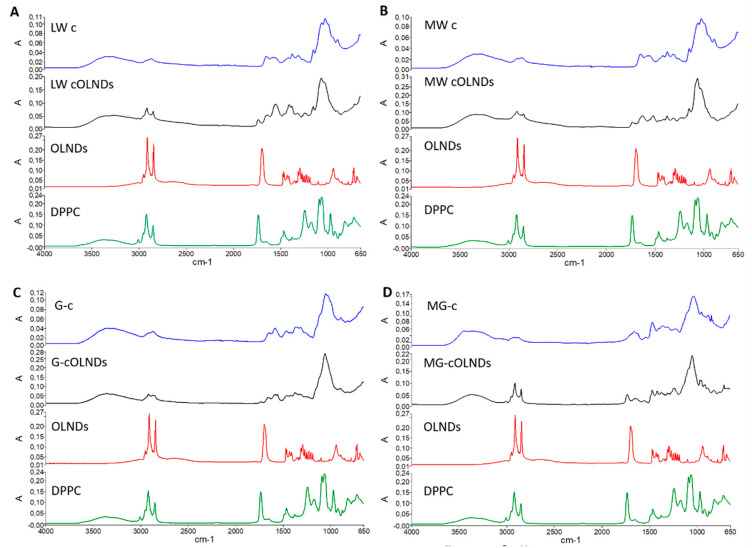
FTIR spectra of chitosan-shelled OLND formulations compared to free chitosan, non-shelled OLNDs, and dipalmitoylphosphatidylcholine (DPPC). (**A**) FTIR spectra of LW chitosan, LW cOLNDs, non-shelled OLNDs, and DPPC; (**B**) FTIR spectra of MW chitosan, MW cOLNDs, non-shelled OLNDs, and DPPC; (**C**) FTIR spectra of G-chitosan, G-cOLNDs, non-shelled OLNDs, and DPPC; (**D**) FTIR spectra of MG-chitosan, MG-cOLNDs, non-shelled OLNDs, and DPPC.

All OLNDs displayed spherical shapes with a core-shell structure, as emerged from TEM analyses. All sizes were in the nanometer range (~400–500 nm as average diameters), with LW cOLNDs being the smallest and MW cOLNDs the largest. All polydispersity indexes were included between 0.19 and 0.24. Zeta potentials ranged from +22.9 (G-cOLNDs) to +40.5 mV (MG-cOLNDs). Oxygen content was similar in all cOLND formulations (~45 mg/L). Figure 3 shows the FTIR spectra of the four chitosan-shelled OLND formulations compared to free chitosan and non-shelled OLNDs. As a control the spectrum of dipalmitoylphosphatidylcholine (DPPC) was also reported.

The characteristic peaks of DPPC at 2857 and 2919 cm^−1^ corresponding to CH2 symmetric and asymmetric stretching vibrations, respectively, and at 1735 cm^−1^ attributed to C = O stretching were clearly detected in the spectrum of non-shelled OLNDs, indicating the presence of the phospholipids on the outer surface of the nanostructure. On the contrary, a shift or disappearance of DPPC peaks were observed for shelled ND formulations, indicating the strong interaction of chitosan with the phospholipid monolayer. Moreover, peak modifications at 1589 cm^−1^ corresponding to the N–H bending of the primary amine were observed among free chitosan and chitosan-shelled OLNDs.

OLND formulations proved to be physically stable over time, as confirmed by long-term checking of their physico-chemical parameters. Indeed, the obtained values did not remarkably change up to six months after the manufacturing of the formulations stored at 4 °C (data not shown). The mucoadhesive properties of cOLND formulations were evaluated by in vitro mucin adhesion assay. The percentage of mucin adhesive to cOLNDs was higher than 80% for all the cOLND formulations, indicating the interaction between cOLNDs and the mucin. The presence of oxygen did not affect cOLND mucoadhesive properties, indeed similar results were obtained with chitosan-coated oxygen-free nanodroplet (cOFND) formulations.

### 2.2. In Vitro Oxygen Release from cOLND Formulations

**Figure 4:**
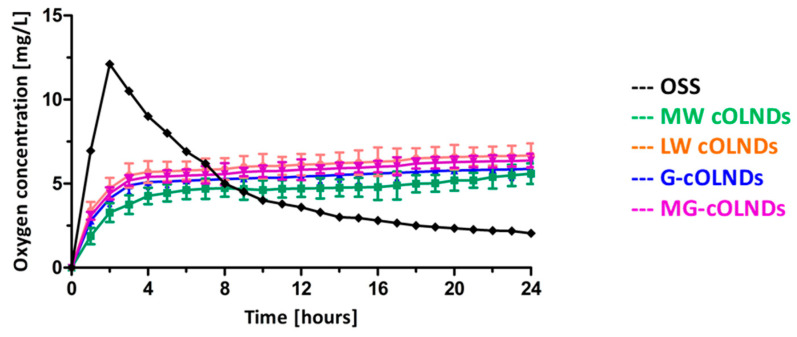
In vitro oxygen release from chitosan-shelled OLNDs and oxygen solution (OSS). LW, MW, G-, and MG-cOLND nanosuspensions as well as OSS were comparatively monitored up to 24 h through an oximeter for oxygen release by diffusion in a hypoxic phase. Results are shown as means ± SD from three independent experiments.

LW, MW, G-, and MG- cOLND as well as oxygen solution (OSS) abilities to release oxygen in vitro were comparatively evaluated by oximetry. Results are shown in Figure 4.

When the nanoformulations were immersed in the hypoxic solution, a prolonged oxygen release profile up to 24 h for all the cOLNDs was observed. There were no significant differences among all four cOLND formulations, proving that they were able to release oxygen through a passive diffusion mechanism, independently from the polysaccharide chosen for the shell. On the contrary, oxygen coming from OSS diffused rapidly in the hypoxic solution, being immediately available, but it led to a great dip-off quite early.

### 2.3. ND Internalisation by Human Keratinocytes

**Figure 5:**
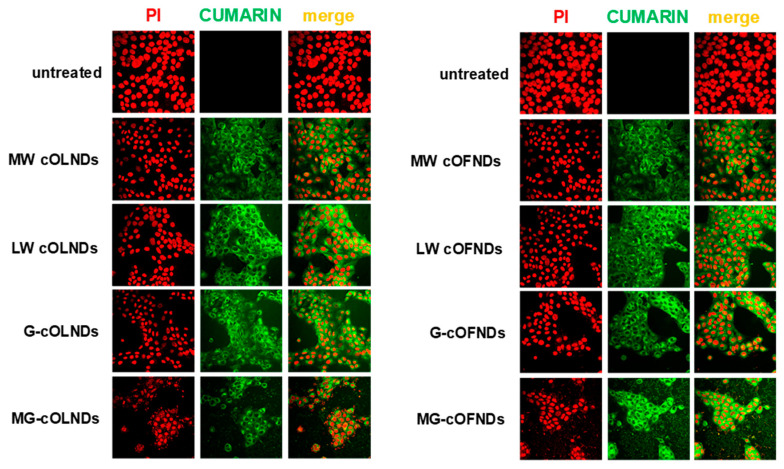
ND internalization by human keratinocytes. Human keratinocyte (HaCaT) cells were left untreated or treated with 10% *v*/*v* 6-coumarin-labeled MW, LW, G-, or MG-chitosan shelled OLNDs (left panels) or oxygen-free nanodroplets (OFNDs) (right panels) for 24 h. After PI staining, cells were checked by confocal microscopy. Results are shown as representative images from three independent experiments. Red: HaCaT cell nuclei after PI staining. Green: 6-coumarin-labeled NDs. Magnification: 60×.

The mechanical interaction between MW, LW, G-, and MG-cOLNDs or cOFNDs and human keratinocytes was investigated through analysis by confocal microscopy. As shown in Figure 5, all ND formulations were avidly internalized into the cytoplasm of human keratinocytes after 24 h of incubation, independently from the type of polysaccharide chosen for the shell and from the presence or absence of oxygen within the core.

### 2.4. Chitosan and ND Biocompatibility with Human Keratinocytes

**Figure 6:**
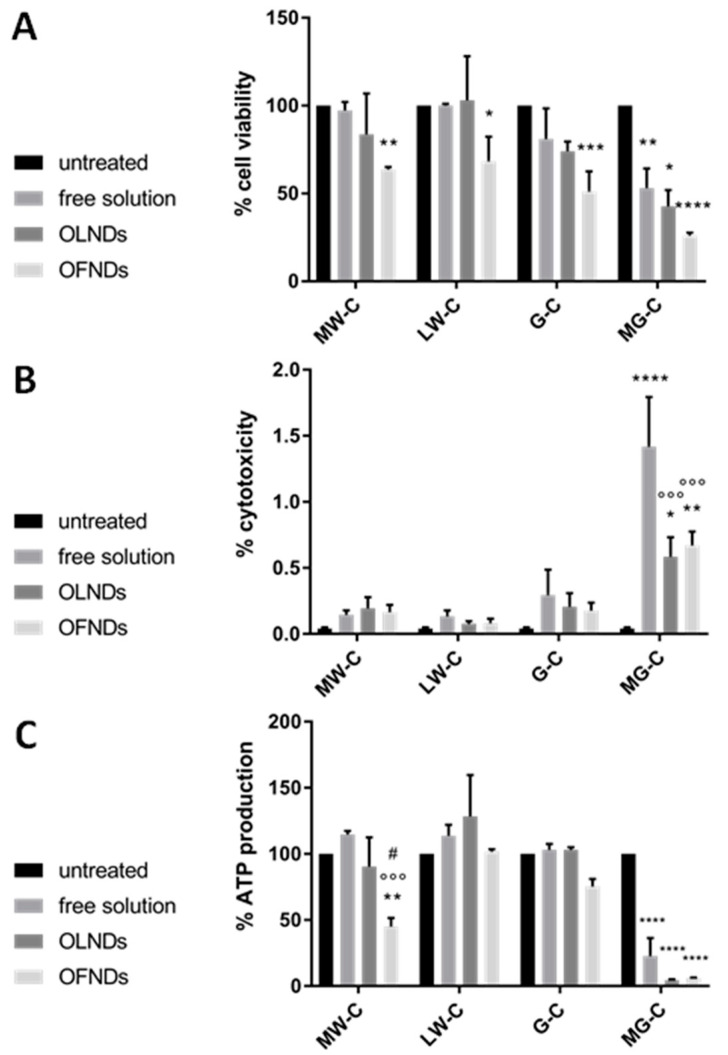
Biocompatibility of chitosan-shelled OLNDs or oxygen-free nanodroplets (OFNDs) with human keratinocytes in vitro. HaCaT cells were left untreated or treated with 10% *v*/*v* MW, LW, G-, or MG-chitosan free solution, cOLNDs or cOFNDs for 24 h. Thereafter, cell viability (**A**), treatment cytotoxicity (**B**), and cell metabolic activity (**C**) were measured through MTT, LDH, and ATP assays, respectively. Results are shown as means ± SEM from three independent experiments. Data were also evaluated for significance by ANOVA. (**A**) Versus untreated cells: * *p* < 0.05; ** *p* < 0.005; *** *p* < 0.0005; **** *p* < 0.0001. (**B**) Versus untreated cells: * *p* < 0.02; ** *p* < 0.01; **** *p* < 0.0001. Versus cells treated with free chitosan solution: °°° *p* < 0.001. (**C**) Versus untreated cells: ** *p* < 0.01; **** *p* < 0.0001. Versus cells treated with free chitosan solution: °°° *p* < 0.0005. Versus cells treated with cOLNDs: # *p* < 0.05.

The biocompatibility of MW, LW, G-, and MG-cOLNDs or cOFNDs as well as free chitosan solutions with human keratinocytes was evaluated over time up to 24 h by using a series of complementary biochemical assays. Specifically, cell viability was checked by MTT assay, treatment cytotoxicity was analyzed by LDH assay, and cell metabolic activity was measured by ATP assay. As shown in Figure 6 (A: MTT; B: LDH; and C: ATP assays, respectively), all the treatments containing MW, LW, or G-chitosan appeared to be highly compatible with viability, health, and metabolism of human keratinocytes.

On the contrary, the treatments containing MG-chitosan appeared to be highly toxic to cells. Interestingly, for each chitosan species or derivative considered, no significant differences in biocompatibility were highlighted between ND formulations (either with or without oxygen in the core) and the free solution counterpart.

## 3. Discussion

Chitin and chitosan have emerged as promising polysaccharides to be employed as candidate biomaterials for the development of new nanomedical devices [39,40]. Chitin, a long-chain polymer composed of repeated units of β-d-(acetylamino)-2deoxy-glucopyranose (*N*-acetyl-d-glucosamine), represents the second ubiquitous natural polysaccharide on Earth after cellulose. This very common polysaccharide can be found either in the exoskeleton or the internal structures of various invertebrates, such as crustaceans and insects [41]. Upon *N*-deacetylation of chitin, chitosan is generated. However, it should be noted that “chitosan” is quite a collective term, as it sums up together a wide spectrum of linear polysaccharides with different molecular weight, viscosity, and degree of deacetylation (ranging from 40% to 98%) [42]. Generally speaking, chitosan can be defined as an amino-polysaccharide composed of randomly distributed β-(1→4)-linked units of deacetylated and acetylated chitin (d-glucosamine and *N*-acetyl-d-glucosamine, respectively). This naturally abundant and renewable cationic polysaccharide displays excellent physico-chemical and biological properties, which are strongly influenced by its molecular weight and degree of deacetylation [43]. Chitosan stands out for its strongly basic nature, due to the protonatable amino groups present in the polymer chains [41]. The protonation constant (pKa) of chitosan is of about 6, depending on its molecular weight and degree of deacetylation. For this reason, it is soluble in acidic aqueous solutions [44]. The number of *N*-acetyl groups in chitosan strongly affects its solubility, crystallinity, and viscosity, as well as its optical properties [45]. Furthermore, the reactivity of chitosan depends on the available amino and hydroxyl groups. In particular, chitosan derivatives can be easily obtained by reacting chitosan primary amino groups as well as its primary or secondary hydroxyl groups [44]. Chitosan derivatives have been designed to improve specific properties of native chitosan, such as solubility [46]. Glycol-chitosan (G-chitosan), soluble at physiological pH, represents a paradigmatic example of a soluble chitosan derivative that has been widely exploited either for diagnostic [47] or therapeutic [48,49] applications in the nanomedical field. Chitosan biocompatibility and biodegradability were largely demonstrated [50]. Chitosan species and/or derivatives are also known to exert direct bacteriostatic or fungistatic activity against several microorganisms (including *Staphylococcus* spp., *Streptococcus* spp., and *Candida* spp.) [51]. Therefore, it has been widely exploited for the preparation of drug delivery systems for the treatment of chronic wounds, such as hydrogels, sponge-like dressings, films, and development of novel nanotherapeutics (nanoparticles, nanocomposites, and nanofibers) [52,53]. 

Based on these preconditions, the present work aimed at investigating and selecting the best candidate chitosan for OLND shell manufacturing in order to optimize OLND formulation parameters and stability. Here, four types of chitosan (i.e., LW, MW, G-, and MG-chitosan) were evaluated for the development of fine-tuned and well-characterized formulations in order to design a multivalent oxygen nanoreservoir for future potential treatment of chronic wounds in human patients. LW, MW, G-, and MG-cOLND nanoformulations were produced following a purposely tuned protocol as previously described [27] and were comparatively evaluated for physico-chemical properties, oxygen releasing abilities, and biocompatibility with human keratinocytes. 

The chitosan shell was selected for its intrinsic properties and because it may be suitable for future drug loading, exploiting either chemical conjugation or electrostatic interactions with the functional groups of chitosan [54,55,56].

All chitosan-shelled OLNDs showed spherical morphology characterized by a well-defined core-shell structure. The presence of chitosan as an external coating can allow the oxygen release to be controlled and prolonged. All nanodroplets displayed average diameters lower than 500 nm, except for MW cOLNDs. Specifically, LW cOLNDs showed the smallest average diameters with a value of about 400 nm. This result can be due to the presence of a greater concentration of chitosan polymer at the external ND surface, compared to MW cOLNDs. In any case, all size values are suitable for skin application [57,58]. 

Zeta potential values ranging from +23 (G-cOLNDs) to +41 mV (MG- cOLNDs) were measured. Interestingly, cationic nanocarriers can be suitable for topical treatments as their positive charges interact strongly with the anionic surface of the skin, as demonstrated by Wu and colleagues [59] in a study showing that cationic fluorophore PMI-conjugated PS-NH^3+^ amino functionalized polystyrene latex nanoparticles displayed brighter fluorescence at the skin surface than anionic PMI-conjugated PS-CO_2_-carboxyl-functionalised polystyrene nanoparticles.

Positive values of zeta potential showed the presence of chitosan on the OLND shell surfaces. Indeed, charged polysaccharides can be adsorbed by the phospholipid interfacial monolayer present at the ND interface, as previously shown [60,61]. Chitosan can interact with phospholipids mainly through electrostatic interactions, but also including H-bonding and hydrophobic forces [60,61]. The formation of a tight chitosan/DPPC monolayer on the OLND surface was confirmed by FTIR analysis. The different types of chitosan can markedly influence these interactions and affect the nanostructure at the interface. In addition, the presence of a charged shell can play a key role for system physical stability. Indeed, the surface charge is a crucial parameter to predict the stability of a nanocarrier, as it measures charge repulsion or attraction among particles. Taking into account a cationic polymer, zeta potential values higher than +20 mV are usually required for stability of colloid systems [62]. Interestingly, the cOLND zeta potential values are high enough to avoid ND aggregation phenomena. The physical stability of the formulations was confirmed by additional monitoring of cOLND physico-chemical parameters up to 6 months. G-cOLNDs appeared to be the least stable out of four formulations, showing a slight size increase over time. This result may be due to their lowest zeta potential value. On the contrary, LW cOLNDs displayed the highest stability because they did not show any size modification up to 6 months. 

In addition, all the formulations showed a marked in vitro mucoadhesive activity. It is worth noting that chitosan is a mucoadhesive polymer. This property, due to the capability to establish ionic, hydrogen, and hydrophobic bonds with the negatively-charged mucin, was maintained in all the formulations. This feature can be exploited to enhance the residence time of cOLNDs in the wound area allowing an intimate contact between cOLNDs and skin lesions [63,64].

All the nanoformulations displayed similar oxygen storing capacity (~45 O_2_ mg/L). The oxygen was slowly released from the internal core of the nanoformulations, delivering clinically relevant amounts of oxygen in a time-sustained manner by passive diffusion through the ND shell. This evidence confirmed cOLND suitability for topical treatment of chronic wounds, as normoxia-like phenotypes of hypoxic skin tissues might be restored due to the prolonged oxygen release. We can speculate that the OLNDs can be incorporated in a topical gel formulation for long-lasting oxygen release for wound care.

Clearly, OLND biocompatibility with human skin cells represents a crucial issue, since the potential use in vivo of oxygen nanocarriers might be compromised by their toxicity to eukaryotic cells. Interestingly, LW chitosan as such (especially chitosan with molecular weight <10000 Da) has been reported to be associated with lower toxicity and higher water solubility compared to chitosan molecules characterized by higher molecular weight [65]. Moreover, chitosan antimicrobial activity related to nanocarriers has been reported to depend on the molecular weight and degree of deacetylation of the chitosan employed during formulation manufacturing [66]. To assess OLND biocompatibility and to discern whether any cytotoxic effects might be related to a shell’s chitosan species or derivatives, LW, MW, G-, and MG-chitosan-shelled nanodroplets as well as free LW, MW, G-, and MG-chitosan aqueous solutions were incubated for 24 h with human keratinocytes. Interestingly, the production of keratinocyte matrix metalloproteinases, playing crucial roles during the remodeling phase of wound healing, was demonstrated to be altered by hypoxia. In addition, taking into account that the donor’s age is a key factor [67], the human keratinocyte cell line employed here (HaCaT cell line) was immortalized from a 62-year-old donor, therefore fitting as a reasonable in vitro model for our studies. Biocompatibility was checked through three different yet complementary assays, measuring cell viability, treatment toxicity, and cell metabolic activity. Results from all three studies were consistent, showing that LW, MW, or G-chitosan (either free or as nanodroplet coating) were highly compatible with viability, health, and metabolism of human keratinocytes, whereas MG-chitosan was not. 

The higher cytotoxicity of MG-cOLNDs can be ascribed to the presence of quaternary ammonium groups belonging to MG-chitosan that are protonated at any pH values. 

Further analyses by confocal microscopy were also carried out to discern the mechanisms of interaction between cOLNDs and human cell surfaces (i.e., lack of contact, adhesion, or internalization). According to the obtained results, all nanodroplet formulations were avidly uptaken by HaCaT cells after 24 h of incubation. This evidence is in line with previous results obtained with dextran OLNBs in monkey fibroblastoid kidney cells [25], with dextran OLNDs in human endothelial cells [30], keratinocytes [28], and monocytes [31], and with MW cOLNDs in human keratinocytes [37] and breast cancer cells [38]. Additionally, the internalization of chitosan/cyclodextrin nanoparticles by epithelial cells [68] have been reported. Further studies aimed at clarifying the mechanisms underlying the physical interaction between cOLNDs and human keratinocytes will be performed. Nevertheless, it is legitimate to speculate that after interacting with human cells, nanoparticles might be engulfed in invaginations of the cell membrane and then be internalized by cells through time-, concentration-, and energy-dependent pinocytic processes [69]. Macropinocytosis, clathrin-mediated endocytosis, caveolae-mediated endocytosis, and mechanisms independent of clathrin and caveolin are examples of these uptake mechanisms [70,71]. These processes can activate several intracellular signaling pathways, thus paving the way for different cellular fates, from proliferation and survival to apoptosis and cell death [72,73]. Chitosan intracellular degradation has been suggested to be associated with lysosomes [74]. Specific receptors for chitosan have not been reported to date, suggesting that the mechanism of chitosan’s cellular binding might be a nonspecific electrostatic interaction with the negatively charged cell membrane. Interestingly, keratinocytes have been reported to expose several anionic residues on their membranes, supporting the present data on chitosan-shelled OLND cellular internalization after adhering on cell plasma membrane. Future studies aiming at elucidating the processes underlying cellular internalization and trafficking of nanodroplets will be considered.

## 4. Materials and Methods

### 4.1. Materials and Instruments

Sterile plastics were from Costar (Corning, NY, USA), Jet Biofil (Guangzhou, China), and VWR (Radnor, PA, USA); ethanol (96%) was provided by Carlo Erba (Milan, Italy); soybean lecithin (Epikuron 200^®^) was from Cargill (Hamburg, Germany); the 1–800 Millipore system to obtain ultrapure water was from Millipore (Molsheim, France); Ultra-Turrax SG215 homogenizer was from IKA (Staufen, Germany); the optical microscope XDS-3FL Optika from Ponteramica, Italy; the Philips CM10 electron microscope was from Philips (Eindhoven, Netherlands); the Nanobrook 90Plus Particle Size Analyzer was from Brookhaven (New York City, NY, USA); the Hach Lange LDO oximeter was from Hach Lange (Derio, Spain); the Beckman Coulter Allegra 64R Centrifuge was from Beckman Coulter (Brea, CA, USA); the Orion Model 420A pH Meter was from Thermo Scientific (Waltham, MA, USA); the HaCaT cells were from Cell Line Service GmbH (Eppelheim, Germany); Dulbecco’s Modified Eagle Medium (DMEM) and RPMI 1640 medium were from Invitrogen (Carlsbad, CA, USA); streptomycin was from Cambrex Bio Science (Vervies, Belgium); the humidified CO_2_/air-incubator was from Thermo Fisher Scientific Inc. (Waltham, MA, USA); the LSM710 inverted confocal laser scanning microscope equipped with Plan-Neofluar 63 × 1.4 oil objective was from Zeiss (Oberkochen, Germany); the Synergy HT microplate reader was from Bio-Tek Instruments (Winooski, VT, USA; the CellTiter-Glo^®^ Kit was from Promega (Madison, WI, USA); Prism version 8.0 software was from GraphPad Software (San Diego, CA, USA). 1,1,1,2,3,4,4,5,5,5-Decafluoropentane (purity ~60%), chitosan low molecular weight (degree of deacetylation 75–85%, 50–190KDa), chitosan medium molecular weight (degree of deacetylation 75–85%, 190–310 KDa), glycol chitosan (degree of polymerization ≥400, purity ≥60%), and methyl glycol chitosan (≥70%) were obtained from Sigma–Aldrich (St. Louis, MO, USA). Any additional materials that are not listed here were from Sigma–Aldrich, St. Louis, MO, USA.

### 4.2. Development and Manufacturing of Formulations 

#### 4.2.1. Preparation of OLND and Control Formulations

OLNDs were prepared following a multi-step protocol that was previously designed, patented, and described by the present research group [27]. Briefly, 300 µL of an ethanol solution containing Epikuron^®^ 200 and palmitic acid (1% *w*/*v*) were added to 500 µL of DFP under magnetic stirring to obtain a pre-emulsion. Then, 4.8 mL of ultrapure water was added, and the system was homogenized using an Ultra-Turrax SG215 homogenizer for 2 min at 24,000 rpm. Thereafter, the sample was saturated with O_2_ for 10 min. Finally, to obtain the polymeric NDs, 300 µL of LW, MW, G-, or MG-chitosan aqueous solution (1% *w*/*v*, pH 5.0) was added drop-wise under magnetic stirring. Control formulations (free MW, LW, G-, or MG-chitosan solutions, OFND suspensions, and oxygen-saturated solution (OSS)) were prepared by following shortened versions of the above-described protocol: Briefly, free chitosan solutions were obtained through proper dilution of MW, LW, G-, or MG-chitosan (1% *w*/*v*) in order to reach chitosan concentrations that were equivalent to those contained in ND formulations, whereas OFND formulations were obtained by omitting during ND manufacturing protocol the addition of O_2_. For biological experiments, all formulations were prepared in phosphate buffer saline (PBS) pH 7.4 instead of ultrapure water. For confocal microscopy experiments, fluorescent NDs were obtained by adding 6-coumarin (1 mg/mL) to the DFP core.

#### 4.2.2. Sterilization of the Formulations

Firstly, the sterilization of the glassware and the components was carried out at 121 °C and at a pressure of 2 bar. Subsequently, the formulations were sterilized by exposure to UV-C for 20 min. At the end, UV-C-treated materials were incubated with cell culture RPMI 1640 medium in a humidified CO_2_/air-incubator at 37 °C for up to 72 h, in order to check any microbial contamination via optical microscopy. 

### 4.3. Characterisation of Formulations

#### 4.3.1. Characterization of ND Formulations

The average diameter, polydispersity index, and zeta potential of cOLNDs were acquired by dynamic laser scattering (DLS). The measurements were carried out using a particle size analyzer at a scattering angle of 90° and at 25 °C. ND nanosuspensions were diluted in deionized filtered water before the analyses. The determination of the zeta potential was obtained by placing the diluted ND formulations in the electrophoretic cell, in which a 15-V/cm electric field was applied. The morphology of formulations was observed by transmission electron microscopy (TEM). ND aqueous suspensions were sprayed on a Formwar-coated copper grid and air-dried before analysis. The osmolarity of the ND formulations was determined at 25 °C using a Knauer osmometer, whereas their viscosity was determined at 25 °C using a Ubbelohde capillary viscosimeter (Schott Gerate, Mainz, Germany).

Fourier transformed infrared (FTIR) analysis was performed using a Perkin Elmer Spectrum 100 FT-IR (Perkin Elmer, Branford, CT, USA). The FTIR spectra of the samples were collected in the region 4000^−1^–650^−1^. Data acquisition was carried out using spectrum software version 10.03.05 Perkin Elmer Corporation. 

A DSC/7 differential scanning calorimeter (Perkin–Elmer, Branford, CT, USA) equipped with a TAC 7/DX instrument controller and the Pyris program were used for thermal analysis. Before performing the analyses, the instrument was calibrated with indium for melting point and heat of fusion. A heating rate of 10 °C/min was set in the 25–200 °C range. Analyses were carried out in triplicate on about 20 mg of sample aqueous suspension placed in standard aluminum sample pans for liquids (Perkin–Elmer) under nitrogen purge. An empty aluminum pan was used as the reference standard.

#### 4.3.2. OLND and OSS Oxygen Content 

The oxygen content of OLNDs and OSS was determined by adding known amounts of sodium sulphite and measuring the generated sodium sulphate, according to Reaction (1):

Na_2_SO_3_ + ½ O_2_ → Na_2_SO_4_ (1)

The amount of sodium sulphate was estimated by gravimetric method after the addition of barium chloride. Briefly, an excess of barium chloride was added to the sample under stirring. The formed precipitate was collected on a filter, washed, and, after drying, was weighted.

#### 4.3.3. ND Stability over Time 

The physical stability of NDs was investigated by morphological analysis and by size and surface charge determination over time for up to six months. ND morphology was observed by TEM to confirm the integrity of ND structure.

#### 4.3.4. Mucoadhesion Test

The in vitro mucin adhesion assay was selected to evaluate the ND mucoadhesive properties. For this purpose, the mucin and ND interaction was determined by turbidimetric analysis. The ND nanosuspensions were mixed with a mucin solution (1 mg/mL) at a 1:1 (*v*/*v*) ratio and left under magnetic stirring for 30 min. Then, the sample was centrifuged for 5 min at 20,000 rpm and the transmittance of the supernatant was measured at 500 nm with an UV spectrophotometer (Du730 spectrophotometer, Beckman, Coulter, Fullerton, CA, USA) to determine the amount of free mucin. The concentration of mucin was calculated using an external standard method. A calibration curve in the concentration range between 0.1 to 1 mg/mL was obtained by analyzing the transmittance of aqueous stock standard solutions. The amount of mucin bound to NDs was determined by subtracting the free mucin content in the supernatant from the total amount of mucin.

### 4.4. In Vitro Oxygen Release

The in vitro oxygen release was evaluated by a dialysis method. The cOLND formulations were sealed in a dialysis bag (cut-off 12,000–14,000 Da) immersed in the hypoxic receiving phase (NaCl 0.9% *w*/*v* solution with an oxygen concentration reduced up to 1 mg/L by a N_2_ purge). The concentration of oxygen released from the different cOLND formulations and OSS into the hypoxic solution was measured up to 24 h through Hach Lange LDO oximeter, displaying an accuracy of 0.01 mg/L. Before each measurement, the oximeter was calibrated in air, until stable temperature and humidity conditions were reached.

### 4.5. Human Biocompatibility Studies

#### 4.5.1. Human Keratinocyte Cell Cultures

For the studies presented here we used the HaCaT, a spontaneously transformed line of adult human skin keratinocytes from a 62-year-old Caucasian male donor [75]. Cells, used for the assessment of chitosan and ND biocompatibility, were grown as adherent monolayers in DMEM supplemented with 10% fetal bovine serum, 100 U/mL penicillin, 100 µg/mL streptomycin, and 2 mM/L glutamine at 37 °C in a humidified atmosphere containing 5% CO_2_. Before starting the experiments, cells were washed with PBS1x, detached with trypsin/ethylenediaminetetraacetic acid (0.05/0.02% *v*/*v*), resuspended, and plated with fresh DMEM at standard cell densities, as detailed in the following sections.

#### 4.5.2. ND Internalization by Human Keratinocytes

Confocal microscopy was used to evaluate the ND internalization by human keratinocytes. HaCaT cells (6 × 10^4^ cells/mL in 24-well plates) were divided into untreated or treated with 10% *v*/*v* 6-coumarin-labeled MW, LW, G-, or MG-chitosan-shelled OLND or OFND nanosuspensions for 24 hours under normoxic condition (20% O_2_) in a humidified CO_2_/air-incubator at 37 °C. Then, HaCaT were fixed with 1% paraformaldehyde solution for 15 min at RT and were incubated with 15 µg/mL propidium iodide (PI) to visualize nucleic acids. Confocal images were acquired by using an Olympus IX70 inverted laser scanning confocal microscope, and captured using FluoView 200 software (Olympus America Inc., Melville, NY, USA). Wavelengths of 488 and 568 nm were used to detect NDs or the labeled nuclei, respectively. The acquisition time was 400 ms. 

#### 4.5.3. Human Keratinocyte Cell Viability

Cell viability was evaluated using 3-(4,5-dimethylthiazol-2-yl)-2,5-diphenyltetrazolium bromide (MTT) assay. HaCaT cells (10^5^ cells/mL in 96-well plates) were left untreated or treated with 10% *v*/*v* MW, LW, G-, or MG-chitosan free solution or chitosan-shelled OLND/OFND nanosuspensions. After incubation for 24 h under 5% CO_2_ at 37 °C, the medium was discarded and 20 μL of MTT in PBS (5 mg/mL) were added to the cells for 3 additional hours at 37 °C. After plate centrifugation and cell supernatant discarding, the dark blue formazan crystals were dissolved using 100 μL of sodium dodecyl sulphate (SDS). The absorbance of the resulting solutions was measured at 550 nm using a Synergy HT microplate reader at a reference wavelength of 650 nm. Data were expressed as percentage of viability.

#### 4.5.4. Chitosan and ND Cytotoxicity on Human Keratinocytes

The potential cytotoxic effects of free chitosan solutions and chitosan-shelled ND suspensions were quantitated measuring lactate dehydrogenase (LDH) release from the cells into the extracellular medium. HaCaT cells (1.5 × 10^5^ cells/mL in 6-well plates) were left untreated or treated with 10% *v*/*v* MW, LW, G-, or MG-chitosan solutions or chitosan-shelled OLND/OFND nanosuspensions for 24 hours under normoxic condition at 37 °C. Then, the culture supernatant (1 mL) was harvested and centrifuged at 13,000× *g* for 30 min. Cells were washed with fresh medium, detached with scraper, washed with PBS 1×, resuspended in 1 mL of 82.3 mM triethanolamine solution, pH 7.6 (TRAP), and sonicated on ice with a 10 s burst. Then, 5 μL of cell lysates and 50 μL of cell supernatants were diluted with TRAP supplemented with 0.5 mM sodium pyruvate and 0.25 mM nicotinamide adenine dinucleotide reduced form (NADH). The Synergy HT microplate reader was used to monitor the reaction, measuring absorbance at 340 nm (37 °C). The intracellular and extracellular LDH activities, expressed as μmol of oxidized NADH/min/well, was determined and the cytotoxicity was eventually calculated as the net ratio between extracellular and total (intracellular + extracellular) LDH activities.

#### 4.5.5. ATP Production by Human Keratinocytes

The number of metabolically active cells in culture was evaluated by determining the levels of adenosine triphosphate (ATP) secreted by the cells into the culture medium. HaCaT cells (6 × 10^4^ cells/mL in 24-well plates) were left untreated or treated with 10% *v*/*v* MW, LW, G-, or MG-chitosan solutions or cOLND/cOFND nanosuspensions for 24 h in a humidified CO_2_/air-incubator at 37 °C. Then, cell supernatants were collected and ATP levels were measured by using the CellTiter-Glo^®^ Luminescent Cell Viability Assay Kit, following the manufacturer’s instructions [76]. 

### 4.6. Statistical Analysis

At least three independent experiments, each one in duplicate or triplicate, were performed for every investigational study. Results are shown as means ± SD for descriptive results or as means ± SEM for inferential results. Imaging data are shown as representative pictures. Numerical data were also analyzed for significance by a two-way analysis of variance (ANOVA) followed by Tukey’s post-hoc test through GraphPad Prism 8 software (GraphPad, San Diego, CA, USA), with *p* < 0.05 being considered as significant.

## 5. Conclusions

In the present study, four different types of chitosan (LW, MW, G-, and MG-chitosan) were challenged as candidate biopolymers for OLND shell manufacturing. The aim of the work was to develop fine-tuned and well-characterized OLNDs, in order to identify an optimized formulation for topical application in wound healing. cOLNDs were analyzed for their physico-chemical characteristics, oxygen releasing abilities, and biocompatibility as well as interaction with human keratinocytes. All chitosan-shelled nanodroplets displayed spherical morphology, cationic surfaces, and diameters in the nanometer range, with LW cOLNDs being the smallest one. They showed mucoadhesive properties and the capability to store and release oxygen in a sustained manner. After cellular internalization, MG-cOLNDs proved to be highly toxic to human keratinocytes, whereas all other OLND formulations were well tolerated. Based on these findings, LW chitosan appears to be the best candidate biopolymer for OLND shell manufacturing, as it associates with the smallest sizes, the highest stability, and the lowest toxicity of nanodroplets. For these reasons, LW chitosan should be employed as the polysaccharide of choice in any future studies aimed at testing the effectiveness of cOLNDs as potential skin devices to treat chronic wounds.

## Figures and Tables

**Table 1:** Physico-chemical characterization of chitosan-coated oxygen-loaded nanodroplets (cOLNDs). LW, MW, G-, and MG-cOLNDs were characterized for average diameter, polydispersity index, and zeta potential by dynamic light scattering. Osmolarity and viscosity were measured using an osmometer and an Ubbelohde capillary viscosimeter, respectively. Results are shown as mean ± SD from ten preparations. See also Figure 1 for further details on nanodroplet structure and morphology.

**Chitosan**	Average Diameter (nm ± SD)	Polydispersity Index ± SD	Zeta Potential (mV ± SD)	Osmolarity (mOsm ± SD)	Viscosity (cP ± SD)
LW	418.2 ± 22.3	0.20 ± 0.02	+32.4 ± 3.5	282 ± 0.6	1.32 ± 0.01
MW	502.1 ± 16.8	0.19 ± 0.01	+30.2 ± 2.8	284 ± 0.5	1.38 ± 0.02
G-	437.2 ± 23.7	0.22 ± 0.01	+22.9 ± 5.4	285 ± 0.4	1.35 ± 0.02
MG-	456.4 ± 27.4	0.24 ± 0.02	+40.5 ± 3.7	290 ± 0.8	1.34 ± 0.02

## References

[1] Hong W.X., Hu M.S., Esquivel M., Liang G.Y., Rennert R.C., McArdle A., Paik K.J., Duscher D., Gurtner G.C., Lorenz H.P. (2014). The role of hypoxia-inducible factor in wound healing. Adv. Wound Care (New Rochelle).

[2] Bowers S., Franco E. (2020). Chronic Wounds: Evaluation and Management. Am. Fam. Physician.

[3] Gurtner G.C., Chapman M.A. (2016). Regenerative medicine: Charting a new course in wound healing. Adv. Wound Care.

[4] Goldberg S.R., Diegelmann R.F. (2020). What Makes Wounds Chronic. Surg. Clin. N. Am..

[5] Zhao R., Liang H., Clarke E., Jackson C., Xue M. (2016). Inflammation in chronic wounds. Int. J. Mol. Sci..

[6] Rousselle P., Braye F., Dayan G. (2019). Re-epithelialization of adult skin wounds: Cellular mechanisms and therapeutic strategies. Adv. Drug Deliv. Rev..

[7] Pitocco D., Spanu T., Di Leo M., Vitiello R., Rizzi A., Tartaglione L., Fiori B., Caputo S., Tinelli G., Zaccardi F. (2019). Diabetic foot infections: A comprehensive overview. Eur. Rev. Med. Pharmacol. Sci..

[8] Kimmel H.M., Grant A. (2016). Ditata, J. The Presence of Oxygen in Wound Healing. Wounds.

[9] Han G., Ceilley R. (2017). Chronic Wound Healing: A Review of Current Management and Treatments. Adv. Ther..

[10] Tejada S., Batle J.M., Ferrer M.D., Busquets-Cortés C., Monserrat-Mesquida M., Nabavi S.M., Del Mar Bibiloni M., Pons A., Sureda A. (2019). Therapeutic Effects of Hyperbaric Oxygen in the Process of Wound Healing. Curr. Pharm. Des..

[11] Dissemond J., Kröger K., Storck M., Risse A., Engels P. (2015). Topical oxygen wound therapies for chronic wounds: A review. J. Wound Care.

[12] Dietz I., Jerchel S., Szaszak M., Shima K., Rupp J. (2012). When oxygen runs short: The microenvironment drives host-pathogen interactions. Microbes Infect..

[13] Hadanny A., Meir O., Bechor Y., Fishlev G., Bergan J., Efrati S. (2016). The safety of hyperbaric oxygen treatment-retrospective analysis in 2,334 patients. Undersea Hyperb. Med..

[14] Gottrup F., Dissemond J., Baines C., Frykberg R., Jensen P.Ø., Kot J., Kröger K., Longobardi P. (2017). Use of Oxygen Therapies in Wound Healing. J. Wound Care.

[15] Bai Q., Han K., Dong K., Zheng C., Zhang Y., Long Q., Lu T. (2020). Potential Applications of Nanomaterials and Technology for Diabetic Wound Healing. Int. J. Nanomed..

[16] Cavalli R., Akhter A.K., Bisazza A., Giustetto P., Trotta F., Vavia P. (2010). Nanosponge formulations as oxygen delivery systems. Int. J. Pharm..

[17] Femminò S., Penna C., Bessone F., Caldera F., Dhakar N., Cau D., Pagliaro P., Cavalli R., Trotta F. (2018). α-Cyclodextrin and α-Cyclodextrin Polymers as Oxygen Nanocarriers to Limit Hypoxia/Reoxygenation Injury: Implications from an In Vitro Model. Polymers.

[18] Shende P., Jain S. (2019). Polymeric nanodroplets: An emerging trend in gaseous delivery system. J. Drug Target..

[19] Krafft M.P. (2020). Alleviating tumor hypoxia with perfluorocarbon-based oxygen carriers. Curr. Opin. Pharmacol..

[20] Bhattacharya D., Ghosh B., Mukhopadhyay M. (2019). Development of nanotechnology for advancement and application in wound healing: A review. IET Nanobiotechnol..

[21] Das S., Baker A.B. (2016). Biomaterials and Nanotherapeutics for Enhancing Skin Wound Healing. Front. Bioeng. Biotechnol..

[22] Alberti T., Coelho D.S., Voytena A., Pitz H., de Pra M., Mazzarino L., Kuhnen S., Ribeiro-do-Valle R.M., Maraschin M., Veleirinho B. (2017). Nanotechnology: A Promising Tool Towards Wound Healing. Curr. Pharm. Des..

[23] Blanco-Fernandez B., Castaño O., Mateos-Timoneda M.Á., Engel E., Pérez-Amodio S. (2020). Nanotechnology Approaches in Chronic Wound Healing. Adv. Wound Care (New Rochelle).

[24] Bisazza A., Giustetto P., Rolfo A., Caniggia I., Balbis S., Guiot C., Cavalli R. Microbubble-mediated oxygen delivery to hypoxic tissues as a new therapeutic device. Proceedings of the 2008 30th Annual International Conference of the IEEE Engineering in Medicine and Biology Society.

[25] Cavalli R., Bisazza A., Rolfo A., Balbis S., Madonnaripa D., Caniggia I., Guiot C. (2009). Ultrasound-mediated oxygen delivery from chitosan nanobubbles. Int. J. Pharm..

[26] Cavalli R., Bisazza A., Giustetto P., Civra A., Lembo D., Trotta G., Guiot C., Trotta M. (2009). Preparation and characterization of dextran nanobubbles for oxygen delivery. Int. J. Pharm..

[27] Magnetto C., Prato M., Khadjavi A., Giribaldi G., Fenoglio I., Jose J., Gulino G.R., Cavallo F., Quaglino E., Benintende E. (2014). Ultrasound-activated decafluoropentane-cored and chitosan-shelled nanodroplets for oxygen delivery to hypoxic cutaneous tissues. RSC Adv..

[28] Prato M., Magnetto C., Jose J., Khadjavi A., Cavallo F., Quaglino E., Panariti A., Rivolta I., Benintende E., Varetto G. (2015). 2H,3H-decafluoropentane-based nanodroplets: New perspectives for oxygen delivery to hypoxic cutaneous tissues. PLoS ONE.

[29] Prato M., Khadjavi A., Magnetto C., Gulino G.R., Rolfo A., Todros T., Cavalli R., Guiot C. (2015). Effects of oxygen tension and dextran-shelled/2H,3H-decafluoropentane-cored oxygen-loaded nanodroplets on secretion of gelatinases and their inhibitors in term human placenta. Biosci. Biotechnol. Biochem..

[30] Basilico N., Magnetto C., D’Alessandro S., Panariti A., Rivolta I., Genova T., Khadjavi A., Gulino G.R., Argenziano M., Soster M. (2015). Dextran-shelled oxygen-loaded nanodroplets reestablish a normoxia-like pro-angiogenic phenotype and behavior in hypoxic human dermal microvascular endothelium. Toxicol. Appl. Pharmacol..

[31] Gulino G.R., Magnetto C., Khadjavi A., Panariti A., Rivolta I., Soster M., Argenziano M., Cavalli R., Giribaldi G., Guiot C. (2015). Oxygen-loaded nanodroplets effectively abrogate hypoxia dysregulating effects on secretion of MMP-9 and TIMP-1 by human monocytes. Mediat. Inflamm..

[32] Riess J.G., Krafft M.P. (1998). Fluorinated materials for in vivo oxygen transport (blood substitutes), diagnosis and drug delivery. Biomaterials.

[33] Ita K. (2017). Recent progress in transdermal sonophoresis. Pharm. Dev. Technol..

[34] Capece S., Domenici F., Brasili F., Oddo L., Cerroni B., Bedini A., Bordi F., Ester C., Paradossi G. (2016). Complex interfaces in ‘phase-change’ contrast agents. Phys. Chem. Chem. Phys..

[35] Banche G., Prato M., Magnetto C., Allizond V., Giribaldi G., Argenziano M., Khadjavi A., Gulino G.R., Finesso N., Mandras N. (2015). Antimicrobial chitosan nanodroplets: New insights for ultrasound-mediated adjuvant treatment of skin infection. Future Microbiol..

[36] Argenziano M., Banche G., Luganini A., Finesso N., Allizond V., Gulino G.R., Khadjavi A., Spagnolo R., Tullio V., Giribaldi G. (2017). Vancomycin-loaded nanobubbles: A new platform for controlled antibiotic delivery against methicillin-resistant *Staphylococcus aureus* infections. Int. J. Pharm..

[37] Khadjavi A., Magnetto C., Panariti A., Argenziano M., Gulino G.R., Rivolta I., Cavalli R., Giribaldi G., Guiot C., Prato M. (2015). Chitosan-shelled oxygen-loaded nanodroplets abrogate hypoxia dysregulation of human keratinocyte gelatinases and inhibitors: New insights for chronic wound healing. Toxicol. Appl. Pharmacol..

[38] Khadjavi A., Stura I., Prato M., Minero V.G., Panariti A., Rivolta I., Gulino G.R., Bessone F., Giribaldi G., Quaglino E. (2018). ’In vitro’, ’in vivo’ and ’in silico’ investigation of the anticancer effectiveness of oxygen-loaded chitosan-Shelled nanodroplets as potential drug vector. Pharm. Res..

[39] Hassan S., Prakash G., Ozturk A.B., Saghazadeh S., Sohail M.F., Seo J., Dokmeci M.R., Zhang Y.S., Khademhosseini A. (2017). Evolution and clinical translation of drug delivery nanomaterials. Nano Today.

[40] Ribeiro D.M.L., Carvalho Júnior A.R., Vale de Macedo G.H.R., Chagas V.L., Silva L.D.S., Cutrim B.D.S., Santos D.M., Soares B.L.L., Zagmignan A., de Miranda R.C.M. (2019). Polysaccharide-Based Formulations for Healing of Skin-Related Wound Infections: Lessons from Animal Models and Clinical Trials. Biomolecules.

[41] Dutta P.K., Dutta J., Tripathi V.S. (2004). Chitin and chitosan: Chemistry, properties and applications. J. Sci. Industr. Res..

[42] Domard A., Domard M. (2001). Chitosan: Structure-properties relationship and biomedical applications. Polym. Biomater..

[43] Qinna N.A., Karwi Q.G., Al-Jbour N., Al-Remawi M.A., Alhussainy T.M., Al-So’ud K.A., Al Omari M.M.H., Badwan A.A. (2015). Influence of molecular weight and degree of deacetylation of low molecular weight chitosan on the bioactivity of oral insulin preparations. Mar. Drugs.

[44] Periayah M.H., Halim A.S., Saad A.Z.M. (2016). Chitosan: A promising marine polysaccharide for biomedical research. Pharmacogn. Rev..

[45] Younes I., Rinaudo M. (2015). Chitin and chitosan preparation from marine sources. Structure, properties and applications. Mar Drugs..

[46] Zhang J., Xia W., Liu P., Cheng Q., Tahirou T., Gu W., Li B. (2010). Chitosan modification and pharmaceutical/biomedical applications. Mar. Drugs.

[47] Key J., Dhawan D., Cooper C.L., Knapp D.W., Kim K., Kwon I.C., Choi K., Park K., Decuzzi P., Leary J.F. (2016). Multicomponent, peptide-targeted glycol chitosan nanoparticles containing ferrimagnetic iron oxide nanocubes for bladder cancer multimodal imaging. Int. J. Nanomed..

[48] Dumont V.C., Mansur H.S., Mansur A.A.P., Carvalho S.M., Capanema N.S.V., Barrioni B.R. (2016). Glycol chitosan/nanohydroxyapatite biocomposites for potential bone tissue engineering and regenerative medicine. Int. J. Biol. Macromol..

[49] Marano F., Argenziano M., Frairia R., Adamini A., Bosco O., Rinella L., Fortunati N., Cavalli R., Catalano M.G. (2016). Doxorubicin-Loaded Nanobubbles Combined with Extracorporeal Shock Waves: Basis for a New Drug Delivery Tool in Anaplastic Thyroid Cancer. Thyroid.

[50] Singh R., Shitiz K., Singh A. (2017). Chitin and chitosan: Biopolymers for wound management. Int. Wound J..

[51] Kulikov S.N., Lisovskaya S.A., Zelenikhin P., Bezrodnykh E.A., Shakirova D.R., Blagodatskikh I.V., Tikhonov V.E. (2014). Antifungal activity of oligochitosans (short chain chitosans) against some *Candida* species and clinical isolates of *Candida albicans*: Molecular weight-activity relationship. Eur. J. Med. Chem..

[52] Vigani B., Rossi S., Sandri G., Bonferoni M.C., Caramella C.M., Ferrari F. (2019). Hyaluronic acid and chitosan-based nanosystems: A new dressing generation for wound care. Exp. Opin. Drug Del..

[53] Las Heras K., Igartua M., Santos-Vizcaino E., Hernandez R.M. (2020). Chronic wounds: Current status, available strategies and emerging therapeutic solutions. J. Control Release.

[54] Cavalli R., Soster M., Argenziano M. (2016). Nanobubbles: A promising efficient tool for therapeutic delivery. Ther. Deliv..

[55] Donalisio M., Argenziano M., Rittà M., Bastiancich C., Civra A., Lembo D., Cavalli R. (2020). Acyclovir-loaded sulfobutyl ether-β-cyclodextrin decorated chitosan nanodroplets for the local treatment of HSV-2 infections. Int. J. Pharm..

[56] Mazzaccaro D., Ticozzi R., D’Alessandro S., Delbue S., Nano G., Costa E., Argenziano M., Cavalli R., Prato M., Basilico N. (2020). Effect of antibiotic-loaded chitosan nanodroplets on Enterococci isolated from chronic ulcers of the lower limbs. Future Microbiol..

[57] Try C., Moulari B., Béduneau A., Fantini O., Pin D., Pellequer Y., Lamprecht A. (2016). Size dependent skin penetration of nanoparticles in murine and porcine dermatitis models. Eur. J. Pharm. Biopharm..

[58] Salatin S., Lotfipour F., Jelvehgari M. (2019). A brief overview on nano-sized materials used in the topical treatment of skin and soft tissue bacterial infections. Expert Opin. Drug Deliv..

[59] Wu X., Landfester K., Musyanovych A., Guy R.H. (2010). Disposition of charged nanoparticles after their topical application to the skin. Skin Pharmacol. Physiol..

[60] Pavinatto F.J., Caseli L., Oliveira O.N. (2010). Chitosan in nanostructured thin films. Biomacromolecules.

[61] Pavinatto A., Delezuk J.A.M., Souza A.L., Pavinatto F.J., Volpati D., Miranda P.B., Campana-Filho S.P., Oliveira O.N. (2016). Experimental evidence for the mode of action based on electrostatic and hydrophobic forces to explain interaction between chitosans and phospholipid Langmuir monolayers. Colloids Surf. B Biointerfaces.

[62] Sze A., Erickson D., Ren L., Li D. (2003). Zeta-potential measurement using the Smoluchowski equation and the slope of the current-time relationship in electroosmotic flow. J. Colloid Interface Sci..

[63] Sogias I.A., Williams A.C., Khutoryanskiy V.V. (2008). Why is chitosan mucoadhesive?. Biomacromolecules.

[64] Sosnik A., das Neves J., Sarmento B. (2014). Mucoadhesive polymers in the design of nano-drug delivery systems for administration by non-parenteral routes: A review. Prog. Polym. Sci..

[65] Wiegand C., Winter D., Hipler U.C. (2010). Molecular-weight-dependent toxic effects of chitosans on the human keratinocyte cell line HaCaT. Skin Pharmacol. Physiol..

[66] Seyfarth F., Schliemann S., Elsner P., Hipler U.C. (2008). Antifungal effect of high- and low-molecular-weight chitosan hydrochloride, carboxymethyl chitosan, chitosan oligosaccharide and N-acetyl-D-glucosamine against *Candida albicans*, *Candida krusei* and *Candida glabrata*. Int. J. Pharm..

[67] Xia X., Zhao Y., Chen A., Mustoe T. (2001). Differential activation of migration by hypoxia in keratinocytes isolated from donors of increasing age: Implication for chronic wounds in the elderly. J. Investig. Dermatol..

[68] Teijeiro-Osorio D., Remunan-Lopez C., Alonso M.J. (2009). Chitosan/cyclodextrin nanoparticles can efficiently transfect the airway epithelium in vitro. Eur. J. Pharm. Biopharm..

[69] Shang L., Nienhaus K., Nienhaus G.U. (2014). Engineered nanoparticles interacting with cells: Size matters. J. Nanobiotechnol..

[70] Xie S., Tao Y., Pan Y., Qu W., Cheng G., Huang L., Chen D., Wang X., Liu Z., Yuan Z. (2014). Biodegradable nanoparticles for intracellular delivery of antimicrobial agents. J. Control. Release.

[71] Treuel L., Jiang X., Nienhaus G.U. (2013). New views on cellular uptake and trafficking of manufactured nanoparticles. J. R. Soc. Inter..

[72] Ivanov A.I. (2008). Pharmacological inhibition of endocytic pathways: Is it specific enough to be useful?. Meth. Mol. Biol..

[73] Johannes L., Lamaze C. (2002). Clathrin-dependent or not: Is it still the question?. Traffic.

[74] Thibault M., Nimesh S., Lavertu M., Buschmann M.D. (2010). Intracellular trafficking and decondensation kinetics of chitosan-pDNA polyplexes. Mol. Ther.

[75] Boukamp P., Dzarlieva-Petrusevska R.T., Breitkreuz D., Hornung J., Markham A., Fusenig N.E. (1988). Normal keratinization in a spontaneously immortalized aneuploid human keratinocyte cell line. J. Cell. Biol..

[76] Mussano F., Genova T., Petrillo S., Roato I., Ferracini R., Munaron L. (2018). Osteogenic Differentiation Modulates the Cytokine, Chemokine, and Growth Factor Profile of ASCs and SHED. Int. J. Mol. Sci..

